# The Role of Immune Cells in Post-Stroke Angiogenesis and Neuronal Remodeling: The Known and the Unknown

**DOI:** 10.3389/fimmu.2021.784098

**Published:** 2021-12-16

**Authors:** Yinzhong Ma, Shilun Yang, Qianyan He, Dianhui Zhang, Junlei Chang

**Affiliations:** ^1^ Shenzhen Key Laboratory of Biomimetic Materials and Cellular Immunomodulation, Institute of Biomedicine and Biotechnology, Shenzhen Institute of Advanced Technology, Chinese Academy of Sciences, Shenzhen, China; ^2^ Department of Neurology, The First Hospital of Jilin University, Changchun, China

**Keywords:** ischemic stroke, inflammation, immune cells, neurogenesis, angiogenesis

## Abstract

Following a cerebral ischemic event, substantial alterations in both cellular and molecular activities occur due to ischemia-induced cerebral pathology. Mounting evidence indicates that the robust recruitment of immune cells plays a central role in the acute stage of stroke. Infiltrating peripheral immune cells and resident microglia mediate neuronal cell death and blood-brain barrier disruption by releasing inflammation-associated molecules. Nevertheless, profound immunological effects in the context of the subacute and chronic recovery phase of stroke have received little attention. Early attempts to curtail the infiltration of immune cells were effective in mitigating brain injury in experimental stroke studies but failed to exert beneficial effects in clinical trials. Neural tissue damage repair processes include angiogenesis, neurogenesis, and synaptic remodeling, etc. Post-stroke inflammatory cells can adopt divergent phenotypes that influence the aforementioned biological processes in both endothelial and neural stem cells by either alleviating acute inflammatory responses or secreting a variety of growth factors, which are substantially involved in the process of angiogenesis and neurogenesis. To better understand the multiple roles of immune cells in neural tissue repair processes post stroke, we review what is known and unknown regarding the role of immune cells in angiogenesis, neurogenesis, and neuronal remodeling. A comprehensive understanding of these inflammatory mechanisms may help identify potential targets for the development of novel immunoregulatory therapeutic strategies that ameliorate complications and improve functional rehabilitation after stroke.

## Introduction

Stroke is one of the leading causes of mortality, with many survivors suffering from long-term disability (1). Results from the 2016 iteration of the Global Burden of Diseases, Injuries, and Risk Factors Study, stroke remained to be the second cause of death globally after ischemic heart disease (1). The financial burden of stroke treatment and prognosis care is heavy. According to the American Heart Association and The American Stroke Association, the total direct medical cost for stroke will rise up to $184.1 billion for the year of 2030 (2). Stroke occurs due to the interruption of blood supply to the brain and may be classified as ischemic or hemorrhagic. Acute ischemic stroke accounts for approximately 87% of all cases of stroke (3). Reperfusion therapy is the most effective treatment for patients with ischemic stroke during the acute phase. However, more than 90% of ischemic stroke patients are unable to receive reperfusion therapy owing to the strict time window (3.5 to 4.5 hours for thrombolysis and 6 hours for thrombectomy). Even for patients receiving reperfusion therapy, more than 40% still suffer from severe complications and develop long-term disabilities, such as language disorders, hemiparesis, cognitive deficits, and dependence on daily activities (4). Unfortunately, due to restricted access to medical resources, high cost and limited regenerative capacity within central nervous system (CNS), neurological rehabilitation, which is considered the main therapy for stroke recovery, can only benefit a small proportion of stroke patients (5). Therefore, understanding the biological processes and pathogenesis of brain injury in the subacute and chronic recovery phase of stroke (>2–3 days) is of critical importance for developing new therapies to improve clinical outcomes of persons with stroke.

Recruitment of leukocytes is an ongoing process that plays an important role in the pathogenesis of ischemic stroke. In addition to accelerating and expanding tissue damage, the inflammatory cascade may also aggravate or alleviate the ischemic insult to the brain (6). Unlike pathogen-associated molecular patterns (PAMPs) which exist in exogenous microbes and drive inflammation in response to infections, damage-associated molecular pattern molecules (DAMPs) are endogenous cell-derived and initiate immunity in response to trauma, ischemia, and tissue damage. In the acute stage of an ischemic stroke, ischemic injury causes the release of DAMPs, such as HMGB1, S100 proteins and heat shock proteins, and subsequently initiates a rapid innate immune response involving infiltrating leukocytes and resident glia cells through Toll-like receptors (TLRs) (7). Initial ischemic injury causes the upregulation of integrin in leukocytes and the associated adhesion molecules on endothelia cells (ECs). Circulating leukocytes are attracted and adhere to the endothelium before being activated by chemokines. Neutrophils are the earliest infiltrating peripheral immune cells, displaying a substantial increase within a few hours after stroke and remaining for up to a week after the initial ischemic insult. Successively, peripheral monocytes, dendritic cells, natural killer (NK) cells, T lymphocytes, and B lymphocytes penetrate the blood-brain barrier (BBB) and infiltrate into the ischemic parenchyma, inducing microglia and astrocyte over-reactivity. These events are part of the pro-inflammatory response and have been excellently reviewed and recently reported in detail (6, 8, 9).

Due to the complex influence of the microenvironment and persistent immune cell infiltration, inflammation may exert both beneficial and harmful effects on the pathogenesis and prognosis of ischemic brain injuries. Previous observations suggest that targeting stroke-related neuroinflammation could become an effective adjunct therapy, but this approach requires caution regarding the timing and avoidance of adverse effects (10, 11). In the subacute and chronic phase of stroke, the transformation of immune cells into alternative phenotypes may provide a neuroprotective effect through the resolution of inflammation and therefore partially re-establishing neurological functions. More importantly, several types of immune cells such as monocytes/macrophages, microglia, and T lymphocytes are also involved in angiogenesis through divergent mechanisms after a stroke occurs. Neurogenesis is traditionally considered the primary target of regenerative strategies for stroke rehabilitation; however, restoration of lost neurons and the entire neurovascular unit is required to achieve a significant structural and functional recovery after ischemic stroke. Therefore, the participation of immune cells in post-stroke rehabilitation may also play an indispensable role in stimulating angiogenesis and further promoting the nerve tissue repair process.

Due to its apparent inflammatory damage in the acute phase and its possible beneficial effects on neural tissue repair processes, the long-term effects of the immune response in stroke remain controversial. In this review, we summarize the relationship between angiogenesis and neuronal remodeling after stroke. We focus on how immune cells participate in the subacute and chronic phases of stroke. This review aims to clarify the roles of different types of immune cells in stroke recovery and potentially enable better stroke treatment through immune regulation.

## Neural Tissue Repair Process After Stroke

Neural tissue repair after the acute phase of stroke involves two main processes: angiogenesis and neuronal remodeling, which further includes neurogenesis and synaptic remodeling (12). After stroke, hypoxia activates ischemic penumbra tissue to release angiogenic factors to increase vascular permeability and establish collateral circulation, and finally connect newly formed blood vessels to the preexisting vascular network. Meanwhile, endogenous neural stem cells (NSCs) initiate proliferation, migration, and differentiation to integrate into the damaged neural circuits. Extensive studies in developmental biology have shown the delicate wiring between newborn microvascular and axonal outgrowths. In pathological states, angiogenesis has been shown to promote neural tissue repair through facilitating neurogenesis and synaptic initiation (12). On the one hand, the factors released by endothelial cells (ECs) promote the differentiation of NSCs and maturation of newborn neurons. In contrast, the main migration process of the neuroblasts is closely guided by blood vessels, which interact to provide direction for neuronal differentiation for NSCs (13). Therefore, it is suggested that the modulation of angiogenesis and neurogenesis affect one another in the process of nerve repair.

### Angiogenesis After Stroke

Ischemic stroke is mainly caused by cerebral vascular stenosis or occlusion. The focal blood flow declines sharply, leading to a shortage in the supply of oxygen and nutrients, eventually causing cell death and tissue damage. The main way that ischemic brain tissue compensates for insufficient oxygen supply is by building new vessels. Angiogenesis in the infarcted area determines the recovery of cerebral blood flow, neuronal regeneration, and reconstruction of synaptic connections between nerve cells, which all influence the degree of functional recovery of the patient (14). The collateral circulation in the acute and subacute phases of ischemia relies on the opening of a pre-existing vascular network, while in the chronic recovery stage, it mainly depends on the formation of new blood vessels. New blood vessels have been shown to appear on the third day and persist for at least 90 days after stroke onset (15, 16). Angiogenesis is stimulated by massive production of VEGF from hypoxic tissues after stroke (17, 18). Proteolytic enzymes, angiogenic growth factors, and inhibitors collaborate to maintain the migration and proliferation of ECs. Vascular endothelial growth factor-A (VEGF-A) has been shown to be significantly elevated, which causes ECs to proliferate and protrude filopodia by coordinating with Notch family receptors and their ligands. The migration and proliferation of ECs are then enhanced and mediated by the interaction between VEGF-A and VEGFR-2 (15, 19). The activation of VEGFR-2 also induces the release of matrix metalloproteinases (MMPs) and endothelial growth factors (EGFs), which may contribute to endothelial progenitor cell migration and basement membrane degradation (20). Previous studies have identified that various immune cells and cytokines are directly or indirectly involved in the regulation of angiogenesis. The cytokines associated with angiogenic effects are listed in **Table 1**.

**Table 1:** The role of pro-inflammatory cytokines in angiogenesis after stroke.

Cytokine	Summary of effects	Citation
MCP-1	Upregulates the expression of VEGF in ECs	(21)
TNF-α	Promotes angiogenesis through VEGF signaling in ECs	(22, 23)
	Promotes EC migration by interacting with TNFR2 and activating Bmx/Etk signaling	
TGF-β	Promotes the proliferation of fibroblasts and ECs	(24, 25)
G-CSF	Promotes the proliferation and migration of ECs	(26, 27)
	Increases the vasal branch points, vascular surface area, and length of blood vessels in the ischemic penumbra region	(28)
FGF	Promotes the proliferation and migration of ECs	(29, 30)
IL-1β	Promotes the migration and proliferation of ECs	(31)
	Inhibits proliferation through VCAM1	(32)
IL-4	Stimulates the phosphorylation of STAT3 and early	(33)
	transcriptional activation of angiogenesis relative genes	
IL-6	Promotes the expression of VEGF	(34, 35)

MCP, monocyte chemoattractant protein; EC, endothelial cell; TNF, tumor necrosis factor; TGF, transforming growth factor; VEGF, Vascular endothelial growth factor; G-CSF, granulocyte colony-stimulating factor; FGF, fibroblast growth factor; IL, interleukin; VCAM1, vascular cell adhesion molecule 1; STAT, signal transducer and activator of transcription.

### Neuronal Remodeling After Stroke

Post-stroke neuronal remodeling is mainly caused by residual neurons that survive ischemia-reperfusion injury and newborn neurons from neurogenesis. Axonal sprouting from residual neurons does not normally appear until 14 days after ischemia, even in the ischemic penumbra (36). The occurrence of newly formed cortical circuits can be detected as early as 3 weeks after ischemia, which is associated with functional recovery (36). Factors released by newly formed blood vessels secrete signals, such as VEGF, artemin, and neurotrophins, which are critical components for axonal sprouting (17, 37). In addition, cytokines from the microglia and invading peripheral immune cells activate astrocytes, which promote angiogenesis and synchronize neuronal activity to induce axonal outgrowths and establish new connections (10, 38, 39). These processes constitute neural tissue repair.

The number of neurons that survive ischemia-reperfusion injury is limited, and newly formed neural connections are generated by neurogenesis. Neurogenesis is the process of generating new functional neurons from endogenous NSCs, including proliferation, migration, and differentiation into mature neurons (40). Neurogenesis becomes highly active following ischemia-reperfusion insults in mainly two distinct regions: the subventricular zone (SVZ) of the lateral ventricles and the subgranular zone (SGZ) of the dentate gyrus of the hippocampus (41). In both areas, primitive pluripotent NSCs express glial fibrillary acidic protein (GFAP). The transcription factor, sex-determining region Y-box 2 (Sox2), and Nestin are termed type B cells in the SVZ, or type I cells in the SGZ (42). These cells are largely quiescent in the physiological state, but they can be activated in response to various external stimuli, such as exercise, hypoxia, and ischemia (43, 44). However, neural regeneration is relatively inefficient, as only a limited number of immature neurons integrate into the existing circuitry and mature into functional neurons. Others undergo programmed cell death and are cleared by microglia (45, 46) In addition, overactivation of transit-amplifying cells by factors secreted by immune cells, glial cells, and newborn ECs during pathological states may lead to the exhaustion of the stem cell pool and early termination of neurogenesis.

### The Relationship Between Angiogenesis and Neuronal Remodeling

**Figure 1:**
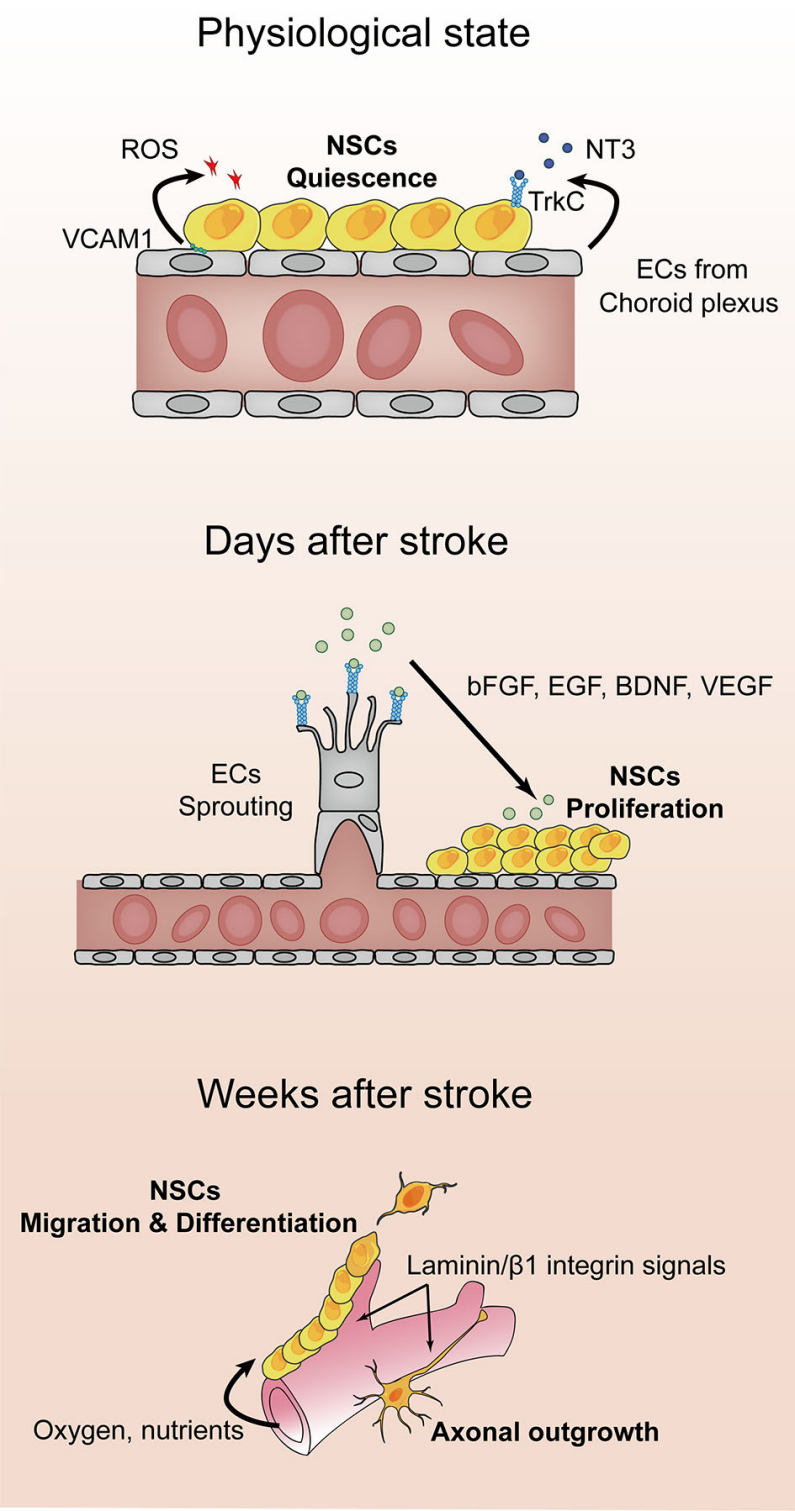
Angiogenesis and neuronal remodeling after stroke. After stroke onset, the hypoxic and ischemic environment triggers vascular sprouting. Vascular endothelial growth factor receptor 2 (VEGFR-2) is expressed on endothelial cells (ECs; shown in gray), where it binds to VEGF-A, which initiates proliferation and protrusion of filopodia. Concomitantly, ECs secrete matrix metalloproteinases and endothelial growth factors to facilitate the migration of endothelial progenitor cells and basement membrane degradation. Microvessels are thought to support neural stem cell proliferation by supplying oxygen, nutrients, and a series of growth factors, including basic fibroblast growth factor, epidermal growth factor, and brain-derived neurotrophic factor. Newly formed blood vessels provide guidance for migration and axonal outgrowth through Laminin/β1 integrin signals.

The microvasculature brings oxygen, nutrients, and growth factors to the area of ischemic injury, which creates an appropriate microenvironment for cell migration. Neuroblasts from the SVZ or SGZ expand and migrate to the peri-infarct region, where post-ischemic angiogenesis occurs. Compelling evidence suggests that several growth factors released from ECs are also indispensable for NSC proliferation, such as basic fibroblast growth factor, brain-derived neurotrophic factor, EGF, and VEGF (47). In addition, angiogenesis promotes vascular-guided NSC migration. Recent studies suggest that Laminin/β1 integrin signals support the synergistic migration of newly formed microvessels and neuroblasts toward injury sites. β1 integrin signals on the surface of microvasculature have been shown to play a critical role in bridging neuroblasts to laminin and promoting cell body movement during migration (48, 49). The relief of hypoxia induced by angiogenesis in the cortex triggers a switch from NSC expansion to neuronal differentiation (50). Together, the factors released by ECs from the nearby microvessels (choroid plexus) are essential for neurogenesis and neuronal maturation during the nerve repair process after stroke (**Figure 1**).

Considering that neurogenesis usually begins over 1 week after stroke onset, infiltrated immune cells and cytokines have been found to affect neurogenesis during this period. Cytokines reported to have potential regulatory effects on NSCs are listed in **Table 2**. Numerous studies have identified a variety of immune cells, such as neutrophils, monocytes, macrophages, microglia, T lymphocytes, and B lymphocytes, that play crucial roles in inflammation-mediated angiogenesis and neurogenesis post-stroke (64, 65). The molecular mechanisms of immune cell-mediated nerve repair in stroke are discussed below.

**Table 2:** The effects of pro-inflammatory cytokines on neurogenesis after stroke.

Cytokine	Summary of effects	Citation
IL-1α	Induces the expression of tyrosine hydroxylase of NSCs	(51)
IL-1β	Promotes astrogliogenesis and reduces the proliferation of NSCs	(52–55)
IL-6	Shifts neurogenesis toward astrogliogenesis	(56, 57)
IL-4	Induces NSCs to differentiate into oligodendrocytes	(58)
IL-10	Maintains NSCs in an undifferentiated state and reduces neuronal differentiation	(59)
IL-17	Promotes the survival and neuronal differentiation of neuroblasts	(60)
IFN-γ	Induces MHC-I expression and upregulates βIII-tubulin	(61, 62)
TNF-α	Promotes astrogliogenesis and inhibits neurogenesis	(52, 56, 61, 63)

NSCs, neural stem cells; IL, interleukin; IFN, interferon; MHC, major histocompatibility complex; TNF, tumor necrosis factor.

## Regulation of Angiogenesis and Neuronal Remodeling by Immune Cells After Stroke

### Neutrophils

Neutrophils are recognized as the first line of defense against pathogens and antigens in the peripheral immune system. As members of the innate immune system, the defense mechanisms of neutrophils have minimal specificity and always cause tissue damage after their activation. Similar to deployed soldiers, neutrophils are fully armed when exiting the bone marrow and require little transcriptional or translational modification to function. Based on these characteristics, neutrophils are the first group of immune cells to infiltrate the brain after ischemic stroke (66). Using *in vivo* two-photon microscopy combined with immunohistochemistry, researchers demonstrated that neutrophils rapidly attach to inflammatory brain ECs within a few minutes and peak at 1–3 days, as seen in permanent and transient middle cerebral artery occlusion (tMCAO) models in mice (67, 68). Neutrophils are characterized by three main functions including phagocytosis, respiratory burst, and formation of neutrophil extracellular traps (NETs). These mechanisms coordinate with one another and cause neuronal and BBB injuries (69). Stimulated by DAMPs, neutrophils release nuclear and granular contents to form a wide network of DNA complexes (NETs), which are used to capture, neutralize, and kill pathogenic microorganisms and prevent their spread. Verified by the expression of Ly6G and protein arginine deiminase 4 (PAD4), Kang et al. found that the formation of intravascular and intraparenchymal NETs peaked at 3–5 days post-stroke in a mice MCAO model (69). PAD4 has been shown to act as an enzyme essential for the NET formation, which contributes to enlarged thrombosis, BBB injury, and reduced neovascularization in the latter stage of stroke (70, 71). Overexpression of PAD4 induces an increase in NET formation, which is accompanied by increased BBB damage and reduced angiogenesis (72). Disruption of NETs by DNase I, inhibition of NET formation by genetic ablation, or pharmacological inhibition of PAD increases revascularization and improves functional recovery. In summary, the above findings suggest that stroke-induced activation of neutrophils form excessive NETs around the BBB and cerebral parenchyma post-stroke, which may affect vascular remodeling during the recovery phase of stroke.

**Figure 2:**
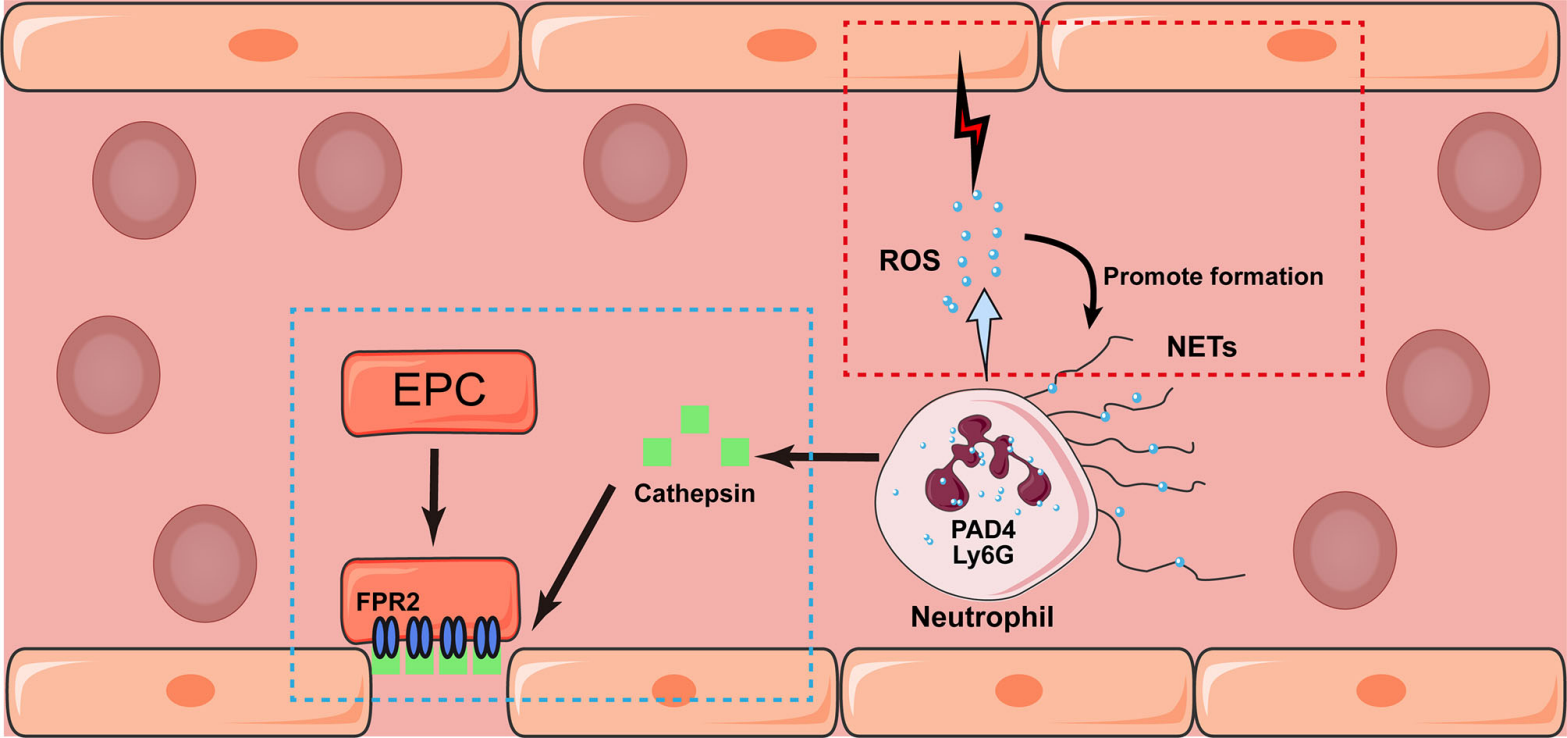
Potential interaction between neutrophil and endothelium after stroke. Neutrophil may exhibit either detrimental (marked in red box) and beneficial (blue box) effects towards the endothelium after stroke. Triggered by phagocytosis, neutrophils release granular contents and formed NETs mediated by the expression of Ly6G and PAD4. In addition, the release of ROS would damage surrounding endothelium and promote the formation of NETs as well. On the contrary, the secretion of Cathepsin would induce the recruitment of circulating endothelial progenitor cell (EPC) in an N-formyl peptide receptor 2 (FPR2)-dependent manner, which promote the angiogenesis in the latter stage of stroke.

Phagocytosis triggers degranulation, including the release of antimicrobial peptides, proteases, and superoxide anions (O^−2^), which act as the “blasting fuse” of reactive oxygen species (ROS). Although ROS plays an important role against pathogens, excess ROS can damage surrounding tissues, such as the BBB, which promotes blood leakage into the brain parenchyma and causes a more severe inflammatory response. Moreover, ROS was shown to mediate the expression of MMP-9 *via* the phosphatidylinositol-3 kinase-mediated signaling pathway, which further impaired BBB integrity (73). Moreover, ROS promotes NET formation by activating PAD4, which induces the unwinding of DNA strands (70). A recent study found that neutrophils mediated the recovery of the endothelium by secretion of Cathepsin, which deposit along the injured arterial lumen induces the recruitment of circulating endothelial progenitor cells in an N-formyl peptide receptor 2-dependent manner in ischemic limb (74, 75). However, only a very small number of neutrophils are present in the brain parenchyma beyond 7 days after stroke onset, which suggests that their function in the latter phase of stroke is mainly secondary to their function in the acute stage (76). In a recent study, skewing neutrophils towards N2 phenotype (CD206^+^) before experimental stroke facilitated the clearance of neutrophils by macrophage, which relief further inflammatory damage and therefore improved long-term recovery (77). The interaction between neutrophils and endothelium are depicted in **Figure 2**.

### Monocytes/Macrophages

#### The Origin and Spatiotemporal Recruitment of Monocytes

The origin of macrophages is complex. Under physiological conditions, monocytes reside in the bone marrow, blood, and spleen in a quiescent state. In response to chemotaxis, circulating monocytes accumulate and infiltrate the injured cerebral parenchyma *via* ECs and differentiate into macrophages during the acute stage of cerebral ischemia. The origin of the monocytes that infiltrate the brain is disputed. The prevailing view is that these monocytes are derived from bone marrow, while a recent study demonstrated that the monocytes that reach ischemic brain tissue originate from the spleen (78). In an observation on the size of the spleen following experimental stroke in rat, it was found that the spleen was significantly decrease from 24 to 48 h and restore at 96 h post stroke. A significant increase of labeled splenocyte (mainly monocytes, T cells and natural killer cells) were found to accumulate around the vasculature in ischemic area in brain at 48 to 96 h post-MCAO (79). The above study provided strong evidence on the origin and spatiotemporal of monocytes following ischemic stroke.

In a retrospective analysis of a single-center database of consecutive thrombolysis cases in acute ischemic stroke, the number of circulating monocytes were substantially increased at 16 days after stroke onset (80). In preclinical evidence of MCAO mice, flow cytometry and immunocytochemistry demonstrated that monocytes are attracted by chemotaxis to the stroke-injured hemisphere and that infiltration appeared within hours and peaked 3 days after stroke. At day 7, half of the infiltrated monocytes/macrophages exhibited a skewed orientation toward the proinflammatory phenotype and the other half toward the anti-inflammatory phenotype. However, during the following 2 weeks, most macrophages exhibited the anti-inflammatory phenotype (81).

Monocyte recruitment is greatly dependent on CCR2, which is expressed in classical monocytes and react with CCL2. Wattananit et al. found that inhibiting monocyte recruitment by an anti-CCR2 antibody in the first week post ischemic stroke prevented long-term behavioral recovery and substantially decreased the expression of anti-inflammatory related genes (81). Using pharmacological inhibition of CCR2 at 1 h before MCAO, and at 2 h and 6 h after MCAO, Chu et al. observed a significant reduction in the number of Ly6C^hi^ monocytes recruited to the brain. At 24 h after MCAO, worse behavioral outcomes and extensive lesions were observed (82). Mice with selective CCR2 deletion in monocytes exhibited an abnormal inflammatory rebound at 15 days post stroke. Moreover, obviously impaired angiogenesis and worse behavioral outcomes were observed, compared with relative wild-type control mice (83). These results suggest that the recruitment of pro-inflammatory monocytes may have both damaging effects in the acute stage of stroke, and protective effects in the chronic recovery stage of stroke. Therefore, it is necessary to carefully consider which anti-inflammatory interventions may adversely affect functional recovery after stroke.

#### The Description of Macrophages Based on Cellular Markers and Morphology

In humans, there are three macrophage subsets: classical CD14^++^CD16^−^, intermediate CD14^+^CD16^+^, and alternative/non-classical CD14^+^CD16^++^. However, there are two main macrophage subsets in mouse blood: classical Ly6C^+^CCR2^hi^CX3CR1^lo^ “inflammatory, classic or M1” macrophages involved in acute inflammation, which are a subset of monocytes first attracted to the ischemic brain tissue, and Ly6C^−^CCR2^lo^CX3CR1^hi^ “alternative, non-classic or M2” macrophages, which may play a role in the repair process. Phenotype by lineage tracing and flow cytometry analysis revealed that the CX3CR1^+^Ly6C^lo^ “repair” macrophages are transdifferentiated from the CCR2^+^Ly6C^hi^ inflammatory macrophages that infiltrate brain tissue in the early phase after ischemia, rather than being independently recruited from the blood (84). However, the regulatory mechanisms underlying the transdifferentiation process remain unclear.

The microenvironment has a significant impact on the morphology of microglia/macrophages. CX3CR1^+^ macrophages exhibited three distinct phenotypes at 14 days and 28 days after MCAO (1): cells with an amoeboid morphology and no plasmalemmal processes, termed amoeboid cells; (2) cells with arborized processes, termed ramified cells; and (3) cells with elongated shapes located along the vessels, termed perivascular cells (85). While the morphology of ramified cells is almost identical to that of microglia, there is a lack of evidence for their functional differences. Amoeboid cells have an extremely fast shape-shifting ability. It is still not clear whether this non-destructive strategy of locomotion is navigated by chemotactic gradients for immune surveillance, whereas these properties are also present in dendritic cells (DCs), despite both being derived from macrophage-DC precursors (86). The perivascular cells exhibited elongated forms along the major axis of the vessel or encircled the entire vessel in the infarcted core and peri-infarcted regions in the MCAO mouse model. Interestingly, through the use of a multi-photon laser to generate a lesion in two endothelial ends in zebrafish, macrophages were shown to adhere to ECs, pull the ruptured ends directly, and narrow the lesion by polymerization of microfilaments through phosphatidylinositol 3- kinase (PI3K)- or Rac1-mediated signaling activation (87).

The description of macrophage activation is currently contentious and confusing, especially for macrophages with an “anti-inflammatory” phenotype in the recovery phase of stroke. Based on the derived source, activators, and a consensus collection of markers, M2 macrophages can be further subdivided into four subtypes: M2a, M2b, M2c, and M2d (88–90). M2a macrophages can be induced by interleukin (IL)-4 and IL-13, while M2b macrophages are induced by the immunoglobulin Fc receptor, lipopolysaccharides, and IL-1β. M2c macrophages are induced by the anti-inflammatory cytokine IL-10 and corticoids, while M2d macrophages are induced by the stimulation of IL-6 and adenosine.

#### The Function of Macrophages After the Acute Phase of Stroke

**Figure 3:**
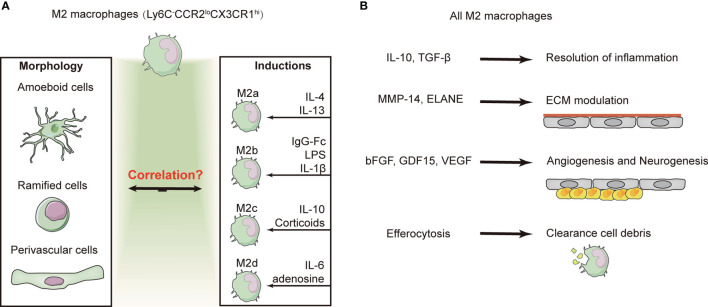
Subtype conversion and function of M2 macrophages. **(A)** Majority of the macrophages exhibit the “alternative/non-classic” subtype (Ly6C^−^CCR2^lo^CX3CR1^hi^) and are characterized as anti-inflammatory at several weeks after ischemic stroke. M2 macrophages exhibit three distinct phenotypes based on their morphology. However, based on the inducers and a consensus collection of markers, M2 macrophages can be divided into four subtypes. The identification of macrophages by morphological or cellular markers remains contentious and confusing. **(B)** Despite the confusion of macrophage identification, overall M2 macrophages have been shown to contribute to the resolution of inflammation, efferocytosis, and angiogenesis.

All four M2 macrophage subtypes express IL-10 and transforming growth factor (TGF)-β with enhanced phagocytosis, which gives them more anti-inflammatory properties (91). In addition, macrophages contribute to nerve repair through efferocytosis and angiogenesis. RNA sequencing analysis of the transcriptome of brain macrophages revealed high activity of biological processes linked to neurovascular remodeling, such as angiogenesis and NSC proliferation (e.g., GDF15, VEGF, and FGF1), as early as 5 days after stroke and lasting for at least 21 days, and peptidases capable of modulating ECM components (e.g., MMP-14 and ELANE) were also upregulated (84). In addition, a large number of efferocytosis-related genes, such as those involved in chemotaxis, recognition of dead cells, engulfment, and processing of phagosomes, were upregulated in brain macrophages at 3–7 days after brain ischemia (92). However, further studies are needed to determine the correlation between the four M2 subtypes with the three different morphologies of CX3CR1^+^ macrophages (**Figure 3**).

### Microglia

There are also a large number of CNS-resident macrophage-like cells, namely microglia, which have phagocytic abilities during tissue damage. Similar to peripheral macrophages which come in with dozens of varieties, microglia also exhibit tremendous heterogeneity, as evidence by advanced transcriptomic and proteomic profiles. Therefore, it is reluctance to paraphrase the microglia with M1/M2 paradigm since the definition are derived from exposing isolated cells to certain stimuli *in vitro*, which were drastic differences from the microenvironment in pathological state *in vivo*, such as tumor, infection, aging, physical trauma and stroke.

Microglia have been previously observed to engulf neutrophils in an ischemic tissue model *in vitro* (93, 94). Subsequently, microglia were also found to activate ECs and capture invasive neutrophils in a cooperative manner (67, 95). Similar to infiltrated macrophages, activated microglia release various pro-inflammatory cytokines, including IL-1β, TNF-α, IL-18, IL-23, and iNOS (96). About 30% of brain resident microglia observed in adult mice are capillary-associated microglia which constantly survey the influx of blood-borne components into the CNS (97). Microglia have been shown to have extensive interaction with ECs and pericytes, which may regulate capillary diameter and cerebral blood flow by altering pericyte or astrocyte coverage (98). In the acute stage of stroke, microglia were attracted to blood vessels with BBB leakage and contributed to the disintegration of blood vessels by phagocytosis of endothelial cells (99). In addition, pericytes were shown to detach from capillary and participate in inflammatory-immunological response mediated by the interaction between DAMPs and TLR4 expressed on the surface of pericytes (100, 101). Interestingly, activated pericytes were found to express microglial markers in both experimental stroke brain and human stroke brain tissue, which may be involved in BBB damage and brain edema (102, 103).

In the chronic recovery stage of stroke, microglia contribute to debris clearance and tissue repair as they engulf newborn neurons that fail to integrate into the neural circuit. Using Rag^-/-^γc^-/-^ mice with ischemic stroke, microglial depletion was shown to exacerbate stroke severity and impair long-term outcomes (104). In contrast, microglial depletion by a CSF1R inhibitor (PLX3397) was also shown to contribute to endogenous neurogenesis and improve functional recovery in a traumatic spinal cord injury model (105). Considered together, both peripheral macrophages and microglia represent a biphasic regulatory mechanism in the context of stroke. Therefore, to achieve therapeutic goals, future studies should focus on elucidating the mechanism of subtype-transdifferentiation rather than simple activation or depletion.

### T Lymphocytes

#### Subpopulations of T Lymphocytes and Their Infiltration

In contrast to myeloid cells (CD18^+^), such as neutrophils and monocytes, T lymphocytes (CD3^+^) are a group of cells with heterogenous subtypes. Both pre-clinical and clinical investigations have revealed that T lymphocyte differentiation is more inclined to pro-inflammatory subtypes in the acute stage of stroke. In patients, circulating T cells were noted from day 1 to at least 124 days after stroke onset, with an expedited increase between days 8 and 20 (106). Preclinically, the earliest time point of T cell infiltration (mostly CD4^+^/GITR^+^) has been reported at 6 h after permanent focal cerebral ischemia (107). However, another study suggested that T-cell infiltration peaks around day 3 in tMCAO and days 5–7 following permanent MCAO (pMCAO) (108).

T cells preferentially accumulate in the peri-infarct area in the acute stage of stroke, and approximately 40% are CD4^+^ helper T cells and approximately 30% are CD8^+^ cytotoxic T cells (109). Depletion of CD4^+^ or CD8^+^T cell subsets by neutralizing antibodies reduced cerebral infarction and relative neurological disorder (110). CD8^+^ cytotoxic T cells are the first T cell subtype to infiltrate ischemic brain tissue and can be observed several hours after stroke onset (109). As lymphocytes that are part of the adaptive immune response, CD8^+^ cytotoxic T cells can cause inflammatory injury to neuronal cells by direct cell contact and secretion of perforin/granzyme after antigen-dependent activation.

In the acute phase of stroke, a multifaceted T cell subtype mediates the release of inflammatory factors. Compared with CD8^+^ cytotoxic T cells, CD4^+^ T cells exhibit more complex phenotypic transformations. Depending on the microenvironment, CD4^+^ T cells may transform into Th1 or Th2 cells, which can acquire both anti-inflammatory and pro-inflammatory effects by secreting various cytokines, including IL-2, IL-12, IFN-γ, IL-4, IL-5, IL-10, and IL-13 (111). Studies on both experimental stroke and human patients with acute ischemic stroke have shown that the cell number of exfoliated ECs, Th17, and circulating γδT cells in patients with acute ischemic stroke were consistent with elevated levels of pro-inflammatory factors, including IL-17A, IL-23, IL-6, and IL-1β (112, 113). Several studies have revealed that IL-17A compromises BBB integrity by reducing the expression of tight junction proteins (TJs), including occludin and ZO-1, in ECs by inducing a robust elevation level of ROS in an NADPH oxidase- or xanthine oxidase-dependent manner (114, 115). In addition, IL-17 has also been shown to facilitate the recruitment of monocytes and neutrophils mediated by CCL2 and CXCL1 expression in ECs (116). Interestingly, IL-17A has also been associated with pro-neurogenesis effects. Determined by qPCR and Western-blot, the expression of IL-17A showed two distinct peaks of expression in the ischemic hemisphere in a tMCAO mouse model: the first occurring within 3 days and the second on day 28 after stroke. IL-17A secreted from reactive astrocytes may augment the survival and neuronal differentiation of neuroblasts from SGZ and SVZ through the activation of p38 MAPK/calpain 1, thereby facilitating synaptogenesis (60).

In the acute stage of stroke, regulatory T cells (Tregs) were found to aggravate the ischemic injury, such as compromising BBB integrity and inducing microvascular dysfunction. However, depletion of Tregs results in alleviation of cerebral tissue damage characterized by increased focal cerebral blood flow and reduced aggregate fibrin (117). The conflicting early effects mediated by Tregs may result from the differences in stroke severity and the immune microenvironment. Although T cells exhibit both detrimental and beneficial immune responses, they account for only a small proportion of the total infiltrating immune cells in the acute phase of stroke.

#### The Central Role of Regulatory T Cells in the Process of Neural Tissue Repair

**Figure 4:**
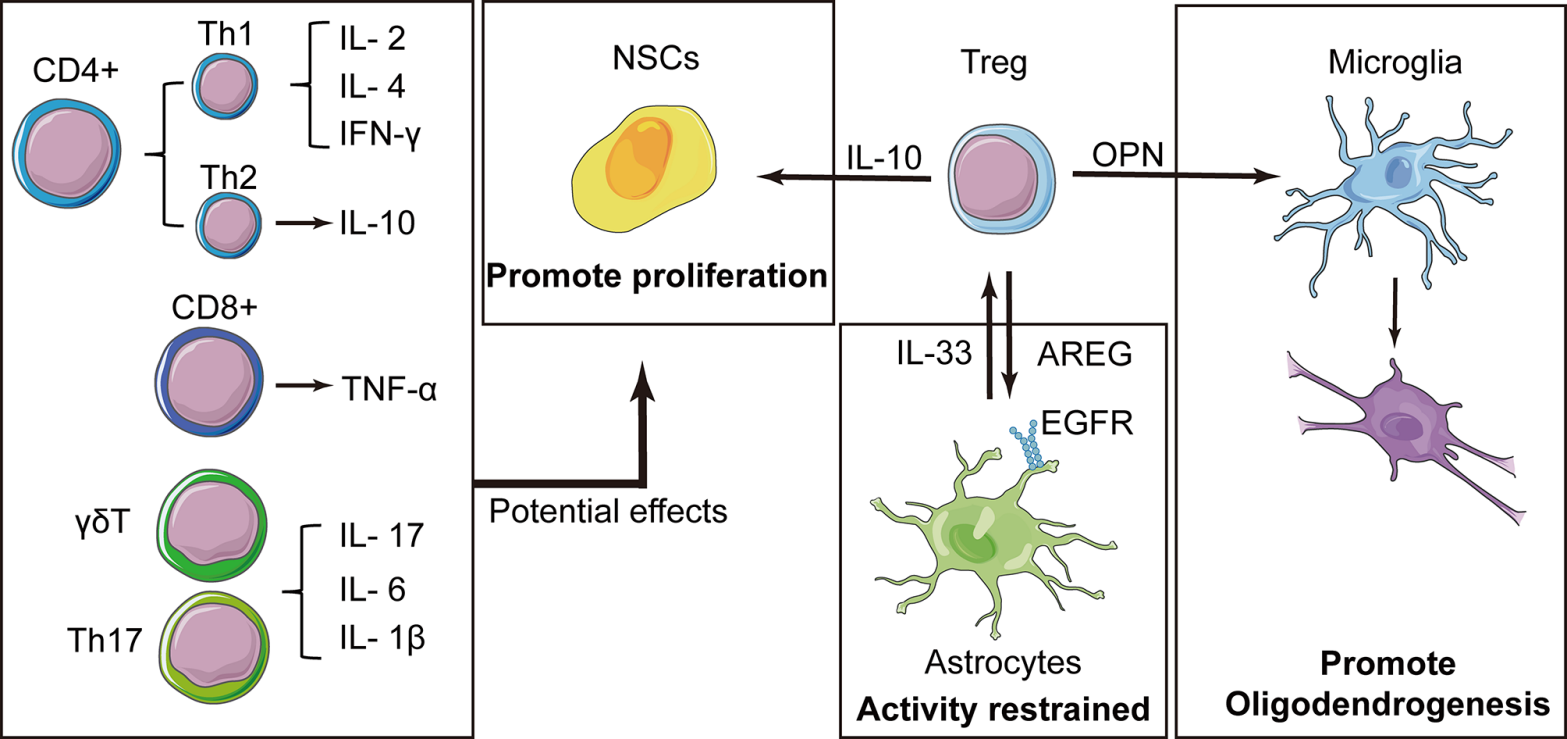
Tregs play a central role in the immune cell-mediated nerve repair process. In addition to the resolution of inflammation, Tregs express interleukin (IL)-10 that contributes to the proliferation of neural stem cells (NSCs). Tregs also produce amphiregulin (AREG), which are bound to epidermal growth factor receptors on astrocytes and inhibit deleterious astrocytic reaction. Osteopontin (OPN) is also secreted by Tregs and induces the reparative phenotype of microglia, promoting oligodendrogenesis. Some cytokines have been reported to have effects on NSCs, and these factors have also been expressed in other types of T cells, suggesting that these T cells may also have potential effects on NSCs.

As an essential subpopulation of immunosuppressive T cells, Treg cells exhibit delayed kinetics of cerebral infiltration, which is associated with multiple protective effects in the chronic recovery stage (more than 1 week) of stroke **(** **Figure 4**). As a minor subpopulation of CD4^+^ T cells, Tregs were identified by a series of markers, including CD25, forkhead box p3 (Foxp3), and Helios (118). Treg cells are best known for their role in sustaining immune homeostasis and restrained inflammatory responses. Moreover, the beneficial roles of Treg cells include pro-remyelination and restraining of astrocytic overreactivity in the late stage of stroke. Previous studies have shown that the levels of circulating Treg cells significantly declined at 2 days post-stroke, which was associated with poor prognosis in human patients (119). Depletion of Treg cells with a CD25-specific antibody causes extensive cerebral tissue injury and elevated expression of inflammatory cytokines, such as TNF-α, IL-1β, and IFN-γ.

IL-10 is a key cytokine involved in the beneficial effects of Tregs, which can downregulate more than 300 genes associated with inflammatory pathways (64). Intraventricular injection of an IL-10 surrogate abolished excessive expression of proinflammatory cytokines after Treg depletion, and prevented secondary infarct growth, whereas the transfer of IL-10-deficient Tregs in an adoptive transfer model was ineffective (120). In addition to anti-inflammatory effects, intraventricular injection of Tregs (1 × 10^5^ cells) was also shown to enhance NSC proliferation in the SVZ of normal and ischemic mice. The number of mammalian achaete-scute homolog 1 (MASH1)-expressing type C cells was decreased by the anti-IL-10 antibody, while GFAP^+^/Nestin^+^ cells were increased at 4 days after stroke, suggesting that an IL-10-mediated neuroprotective effect may facilitate the proliferation of NSCs in the SVZ. However, the underlying mechanism remains elusive (121).

Solid evidence has shown that Foxp3^+^ Tregs have a protective effect on the neuroinflammatory response after stroke (47). Interestingly, Foxp3^+^ Treg cells were also shown to inhibit the activation of astrocytes following cerebral injury. Using a tMCAO model in mice, Ito et al. discovered that at 14 days after stroke, astrocytes and oligodendrocytes attracted the infiltration of blood Tregs into the brain through the signaling of chemokines, CCL1-CCR8 and CCL20-CCR6. Tregs played a critical role in suppressing the over-activation of astrocytes through the expression of amphiregulin, which were bound to epidermal growth factor receptors (EGFR) on astrocytes. In addition, astrocytes facilitate the proliferation of Tregs by releasing IL-33, which in turn downregulates astrocyte activity (117, 122). In a rodent model with amphiregulin -deficient Tregs, astrocytes exhibited excessive activity with significant increases in the expression of IL-6. This exaggerated astrocytic reaction can be mitigated by the replenishment of wild-type Tregs or intraventricular amphiregulin treatment. In another study, depletion of Treg cells by diphtheria toxin increased the number of invading pro-inflammatory T cells (CD3^+^ and CD8^+^), cytokine production, and astrocytosis in response to traumatic brain damage (123).

Besides the direct interactions with astrocytes or NSCs mentioned above, Hu et al. identified a prominent Treg cell cluster (CD4^+^CD25^+^Foxp3^+^) through single-cell RNA sequencing. Tregs showed robust accumulation in the injured brain at days 7–35 after ischemic stroke. Interestingly, adoptive cell transfer of WT Tregs increased the number of newborn APC^+^BrdU^+^ oligodendrocytes in Rag1^-/-^ lymphopenic mice, but this effect was abolished when microglia were depleted. Further analyses revealed that osteopontin released from Treg cells strengthens the phagocytosis of microglia through activation of integrin receptors, which facilitates oligodendrogenesis and white matter regeneration (124).

### B Lymphocytes

While immediate and early proinflammatory responses to stroke are mainly mediated by innate immune cells, B cell-mediated adaptive immune responses exhibit delayed pro-injury functions in the latter stages. The initiation of an adaptive immune response specific for CNS antigens can be observed at around week 4, when primed antigen-presenting cells present cell fragments as antigens to T cells and B cells (125). B cells mainly function in three ways: antigen presentation, antibody production, and cytokine secretion. Naïve B cells express the primary effector antibodies, IgM and IgD. After receiving antigens, B cells initiate isotype transformation, express plasma cell markers, and produce antigen-specific IgG or IgA antibodies, which may be indirectly associated with some stroke-related risk factors, including hypertension, diabetes, and atherosclerosis (126). Stroke may lead to subsequent vascular dementia. In a distal pMCAO model, mice developed short-term memory deficits between weeks 1 and 7 following cerebral infarction. B cells were found to accumulate in the infarct region and secreted IgA and IgG at 4–7 weeks after stroke (127). Genetic deficiency and pharmacologic ablation of B-lymphocytes using an anti-CD20 antibody prevented delayed-onset cognitive deficits, suggesting that immunoglobulin synthesis by B-lymphocytes may be involved in long-term injury to neuronal cells after stroke. In a retrospective cohort study, immunoglobulin synthesis (IgG, IgM, and IgA) in the cerebrospinal fluid of stroke patients was found several months after stroke onset (128). The antibodies may activate the complement pathway and cause further damage to neuronal cells, which is a main cause of neuronal death in multiple sclerosis (129). Concrete evidences have proved that massive memory B cells and antibody-producing plasma blasts were exist in the CSF of patients with multiple sclerosis (130, 131). In addition to the production of IgG, B cell and its subtypes were also found to be involved in the pathogenesis of MS by secretion of proinflammatory cytokines, such as lymphotoxin-alpha, CXCL12, and CXCL13 (132). Therefore, it is reasonable to speculate that B cells may also play a key role in the chronic stage of stroke. Up to date, there is no explicit evidence of the beneficial role of B cells in the chronic stages of stroke. In gene ablation mice models, mice deficient in lymphocytes (Rag^−/−^), or specific depletion of CD4^+^ T cells, CD8^+^ T cells, B cells, or IFN-γ, was used to determine the contribution of different lymphocyte subgroups to ischemia reperfusion injury and recovery. Smaller infarction volumes and amelioration of neurological disorders were found in mice lacking lymphocytes (Rag^−/−^), but no improvements were observed in mice lacking B cells. Furthermore, B cell transfer in Rag^−/−^ mice did not shown any significant improvement, indicating that B cells may not be play a protective role in the chronic stage of stroke (133). On the contrary, B cells may have negative effects on post-stroke neuroprotection. In an MCAO model in μMT^–/–^ mice (B cell depletion), enlarged areas of infarction, neurological disorders, and higher mortality were observed (134, 135). A higher number of invading circulating immune cells, including activated T cells, macrophages, microglial cells, and neutrophils were found in the ischemic hemispheres of μMT^−/−^ mice. It is worth noting that beneficial effects were observed after adoptive transfer of B cells from WT mice into μMT^−/−^ mice prior to MCAO modeling. Interestingly, it is reasonable to speculate that these protective effects were mediated by IL-10 since no obvious change was observed when B cells were transferred into IL-10-deficient mice.

### Other Types of Immune Cells

In addition to the above immune cells, DC and NK cells have also been reported to be involved in the pathological process of stroke. However, their main role is to coordinate with other types of cells to facilitate an immune response. DCs are the most efficient antigen-presenting cells. Under physiological conditions, DCs mainly patrol near the cerebrospinal fluid, such as the meninges and choroid plexus, and are barely present in cerebral parenchyma (136). In a pMCAO model, DCs (OX6^+^) were found to invade the ischemic core within the first few hours and gradually increased until 6 days after ischemia (137). However, in addition to acting as antigen-presenting cells, their functions remain unclear. NK cells are innate lymphocytes that can be swiftly mobilized during the earliest phases of immune responses. Recruited by CXCL8, NK cells have been shown to accumulate in the ischemic hemisphere and aggregate in the peri-infarction area through the release of IFN-γ and ROS in the acute stage of stroke (138, 139). However, the spatiotemporal and phenotypic profiles of NK cells after stroke are yet to be elucidated. In addition to peripheral immune cells, the meningeal immune cells have recently been recognized as a potential immune repertoire which may have an impact on the neuro-immune crosstalk during stroke (140). Study have shown that the meningeal mast cells, including granulocytes and activated macrophages, may contribute to stroke-related pathology in MCAO mice (141). Although the properties of meningeal immunity have yet to be fully elucidated, its potential threat or benefit to the CNS are worth being addressed. The roles of immune cells in angiogenesis, neuronal remodeling, and neurogenesis are summarized in **Table 3**.

**Table 3:** The role of various immune cells on angiogenesis, axonal outgrowth and neurogenesis after stroke.

Immune cells	Angiogenesis	Axonal outgrowth	Neurogenesis
Neutrophils	Reduces the survival of ECs (72)	Promotes axon regeneration (142)	No reports
	Promotes the recruitment of endothelial progenitor cells (143)		
Macrophages	Protects the endothelium (87)	Promotes axonal sprouting by release of GDNF (144)	Promotes migration and proliferation (145)
Microglia	No reports	Promotes axonal sprouting (144)	Reduces the survival of NSCs (146)
			Promotes the proliferation of NSCs (147)
			Promotes the survival of newborn neurons (148)
Treg cells	No reports	Promotes oligodendrogenesis (124)	Promotes the proliferation of NSCs (121)

NSCs, neural stem cells; EC, endothelial cells, GDNF, glial cell-derived neurotrophic factor, Treg, regulatory T cells.

## Discussion

Currently, intravenous thrombolysis with recombinant tissue plasminogen activator and mechanical thrombectomy has achieved exhilarating effects in the treatment of acute ischemic stroke. However, these treatments are still limited by their strict time windows. Neuroprotective therapies targeting neuronal cells for the treatment of acute ischemic stroke are still being developed, and anti-inflammatory treatment remains the conventional method of stroke treatment.

Immune cells have long been considered as acute or chronic sources of inflammation. More recently, the protective roles of various types of immune cells have been discovered, which provides a new prospect for stroke treatment. However, it is difficult to determine when to promote or terminate immune interventions in ischemic stroke owing to the extremely complex spatiotemporal phenotype of immune cells. With the development of single-cell sequencing and space transcriptome technology, we are rediscovering the role of the immune response in stroke with unprecedented depth. Based on current knowledge, multiple types of immune cells are involved in the process of neural tissue repair in the late stage of stroke by means of factor secretion. Therefore, to understand the “Janus Face” of the immune system in the different pathological processes of stroke, detailed studies based on the latest technologies are needed to inform new perspectives on stroke treatment.

## Author Contributions

YM designed and drafted the manuscript. SY participated in the design and writing of this manuscript. JC, QH, and DZ provided constructive advice and edited the manuscript. All authors contributed to the manuscript and approved the submitted version.

## Funding

This work was funded by the National Natural Science Foundation of China (81771293 to JC; 81803528 to YM), the Science Technology and Innovation Commission of Shenzhen (ZDSYS20190902093409851, JCYJ20210324115800003, SGLH20180625142404672), the International collaboration project of Chinese Academy of Sciences (172644KYSB20200045), and the CAS-Croucher Funding Scheme for Joint Laboratories, and Guangdong Innovation Platform of Translational Research for cerebrovascular diseases.

## Conflict of Interest

The authors declare that the research was conducted in the absence of any commercial or financial relationships that could be construed as a potential conflict of interest.

## Publisher’s Note

All claims expressed in this article are solely those of the authors and do not necessarily represent those of their affiliated organizations, or those of the publisher, the editors and the reviewers. Any product that may be evaluated in this article, or claim that may be made by its manufacturer, is not guaranteed or endorsed by the publisher.
